# Gene Expression Analysis of *Plum pox virus* (Sharka) Susceptibility/Resistance in Apricot (*Prunus armeniaca* L.)

**DOI:** 10.1371/journal.pone.0144670

**Published:** 2015-12-11

**Authors:** Manuel Rubio, Ana Rosa Ballester, Pedro Manuel Olivares, Manuel Castro de Moura, Federico Dicenta, Pedro Martínez-Gómez

**Affiliations:** 1 Department of Plant Breeding, Centro de Edafología y Biología Aplicada del Segura (CEBAS-CSIC), PO Box 164, E-30100 Espinardo (Murcia) Spain; 2 Department of Food Science, Instituto de Agroquímica y Tecnología de Alimentos (IATA-CSIC), Avda. Agustín Escardino 7, 46980 Paterna (Valencia) Spain; 3 aScidea Computational Biology Solutions, S.L. Parc de Reserca UAB, Edifici Eureka. 08193 Bellaterra (Cerdanyola del Vallés), Barcelona, Spain; Wuhan Botanical Garden of Chinese Academy of Sciences, CHINA

## Abstract

RNA-Seq has proven to be a very powerful tool in the analysis of the *Plum pox virus* (PPV, sharka disease)/*Prunus* interaction. This technique is an important complementary tool to other means of studying genomics. In this work an analysis of gene expression of resistance/susceptibility to PPV in apricot is performed. RNA-Seq has been applied to analyse the gene expression changes induced by PPV infection in leaves from two full-sib apricot genotypes, “Rojo Pasión” and “Z506-7”, resistant and susceptible to PPV, respectively. Transcriptomic analyses revealed the existence of more than 2,000 genes related to the pathogen response and resistance to PPV in apricot. These results showed that the response to infection by the virus in the susceptible genotype is associated with an induction of genes involved in pathogen resistance such as the *allene oxide synthase*, *S-adenosylmethionine synthetase 2* and the *major MLP-like protein 423*. Over-expression of the *Dicer protein 2a* may indicate the suppression of a gene silencing mechanism of the plant by PPV HCPro and P1 PPV proteins. On the other hand, there were 164 genes involved in resistance mechanisms that have been identified in apricot, 49 of which are located in the *PPVres* region (scaffold 1 positions from 8,050,804 to 8,244,925), which is responsible for PPV resistance in apricot. Among these genes in apricot there are several *MATH* domain-containing genes, although other genes inside (*Pleiotropic drug resistance 9* gene) or outside (CAP, *Cysteine-rich secretory proteins*, *Antigen 5 and Pathogenesis-related 1 protein*; and LEA, *Late embryogenesis abundant protein*) *PPVres* region could also be involved in the resistance.

## Introduction

Sharka, a disease caused by the *Plum pox virus* (PPV), which belongs to the *Potyvirus* genus within the family *Potyviridae*, is the most important disease affecting temperate fruit species including apricot (*Prunus armeniaca* L.) (see recent reviews García et al. [1] and Clemente-Moreno et al. [2]). The use of genetic resistance sources offers the only definitive solution for the control of sharka. Some of these natural sources of resistance to PPV in apricot were first identified in North America [3] and since then have been widely exploited in breeding programmes [4,5]. There are different propositions about the genetic control of resistance to PPV in apricot, involving one, two or three genes [6,7]. In 2007, Rubio et al. [8] analysed several of these propositions in apricot, but none fully fit the phenotypic segregation of the populations studied by these authors. These results reflect the difficulty of establishing accurate genetic control of resistance to PPV.

Regarding the genomic bases of resistance to PPV in apricot, Hurtado et al. [9] used Amplified Fragment Length Polymorphism (AFLP) markers to identify a major Quantitative Trait Locus (QTL) responsible for resistance to PPV, located in linkage group (LG) 1 of apricot. Later, subsequent works using AFLPs and Simple Sequence Repeats (SSRs) also identified this same QTL as well as other QTLs in different regions of LG1 and LG5 [10–18]. In LG1, Pilarová et al. [19] identified two QTLs linked to resistance to Dideron PPV strains and three QTLs linked to Marcus strains. These authors also identified a minor QTL in LG5 related to resistance to Dideron PPV strains. Several molecular markers linked to the QTL of LG1 and PPV resistance have been identified by Dondini et al. [18], Vera-Ruiz et al. [20], and Soriano et al. [21]. All these authors have also indicated that the resistant alleles from the SSRs co-segregate with PPV resistance. More recently, Zuriaga et al. [22] described a 196 kb genomic region in LG1 (scaffold 1 of the peach reference genome, positions 8,050,805–8,244,925) of the syntenic to the *PPVres* locus. Other authors have also described a family of *Meprin* and *TRAF-C homology domain* (*MATH*) genes as candidates for resistance [22,23]. However, recent studies highlight the possibility of a second locus involved in the control of this trait [4,5].

Despite remaining doubts about the genes involved in PPV resistance, molecular studies at the transcriptomic level in the analysis of PPV-apricot interaction are scarce. First approaches involved the partial analysis of candidate genes (CGs) including *Nucleotide binding site-leucine rich repeat* (*NBS-LRR*) genes [12,24,25]; the putative *RNA-helicase SDE3* [14]; *RTM* [*Restricted Tobacco etch virus (TEV) movement*] genes [26]; the *Argonaut AGO1* protein [14]; and the *Eukaryotic translation initiation factor* (*eIF4E*) [17]. The results obtained by all of the authors mentioned above were not conclusive at all regarding the expression of genes involved in resistance to PPV in apricot. The first complete study at the transcriptome level using cDNA-AFLP showed a differential expression of 21 genes after inoculating the resistant genotype ‘Goldrich’ with PPV [27], although there were no clear candidate genes responsible for this resistance. Today, high-throughput gene expression analysis using massive RNA (cDNA) sequencing (RNA-Seq) is the most powerful tool available for characterising transcriptomes [28–29]. Indeed, this tool has the potential to better elucidate the gene expression mechanism associated with PPV resistance in apricot.

RNA-Seq has been used to study the hypersensitive response to PPV in the European plum ‘Jojo’ (*Prunus domestica* L.) [30] and PPV susceptibility in peach [*P*. *persica* (L.) Batsch] [31]. In the first study, a total of 3,020 differentially expressed unigenes were regulated after the PPV inoculation of the resistant plum genotype ‘Jojo’, which developed a hypersensitive response. Out of these unigenes, 154 were characterised as potential resistance genes, and 10 of these could be placed in the *NBS-LRR* class of resistance genes [30]. In the second study, Rubio et al. [31] described a total of 1,554 differentially expressed peach genes after analysing leaves from the ‘GF305’ peach genotype inoculated with PPV. Early PPV infection in leaves without symptoms from PPV-infected peach plants is associated with an induction of genes related to pathogen resistance. On the other hand, when the virus was established, the over-expression of *Dicer protein 2a* genes may indicate the suppression of a gene silencing mechanism of the plant by PPV HCPro and P1 PPV proteins.

To contribute to knowledge in the field, in this study we analysed the gene expression changes in leaves from two full-sib apricot genotypes, resistant (‘Rojo Pasión’) and susceptible (‘Z506-7’), in response to *Plum pox virus* infection using high-throughput Illumina sequencing (RNA-Seq).

## Material and Methods

### Plant material

The plant material consisted of two apricot genotypes, ‘Rojo Pasión’ and ‘Z506-7’, obtained from the cross between the North American cultivar ‘Orange Red’ (resistant to PPV) and the Spanish cultivar ‘Currot’ (susceptible to PPV) [3]. Experiments were performed in controlled greenhouse conditions in the Experimental Field of CEBAS-CSIC at Santomera (Murcia, Spain). ‘Rojo Pasión’ is self-compatible and early blooming and has an intermediate-sized oblong fruit with yellow skin, intense red blush and a light orange flesh colour. This genotype is characterised by its resistance to PPV [32]. ‘Z506-7’ is also self-compatible and early blooming and has a fruit similar to that of ‘Rojo Pasión’ and characterised by its great susceptibility to PPV [5]. In addition, a preliminary characterization using 61 different SSR markers showed a percentage of 72% of SSR alleles in common (S1 Table). Finally, ‘Real Fino’ apricot seedlings, which are very susceptible to PPV, were used as rootstock.

### PPV evaluation procedure

Plant inoculations were carried out in the ‘Real Fino’ rootstocks using the PPV-D strain 3.30 RB/GF-IVIA (GenBank: KJ849228.1). Four replications of two-month-old ‘Real Fino’ seedling rootstocks grown in 3.5L pots were inoculated by grafting a piece of bark from other previously infected ‘Real Fino’ plants showing strong sharka symptoms. Two additional seedlings were kept as controls. Two months later, six replicates of clonal ‘Rojo Pasión’ and ‘Z506-7’ were grafted onto the previously inoculated and control ‘Real Fino’ seedling rootstocks. One month after grafting, plants were subjected to an artificial period of dormancy in darkness at 7°C for two months before being transferring again to the greenhouse. After two months in the greenhouse, sharka symptoms on leaves were scored using a scale from 0 (no symptoms) to 5 (maximum intensity). The presence of PPV was confirmed by DASI-ELISA with the specific monoclonal antibody 5B-IVIA/AMR (Plant Print Diagnostics SL, Valencia, Spain) and RT-PCR analysis using specific primers for the coat protein VP337 (5’ CTCTGTGTCCTCTTCTTGTG 3’) and VP338 (5’ CAATAAAGCCATTGTTGGATC 3’). At this moment, leaves were collected in all of the assays performed: SSR analysis, RNA-Seq and qPCR assay.

### High-throughput RNA sequencing

The apricot samples analysed included ‘Rojo Pasión’ (R, resistant to PPV) and ‘Z506-7’ (Z, susceptible to PPV) apricot cultivars grafted onto healthy (c, control) and PPV inoculated (i, inoculated) ‘Real Fino’ apricot seedling rootstocks. Control replicates did not show sharka symptoms and were ELISA an RT-PCR negative. In the inoculated treatments ‘Z506-7’ replicates showed strong sharka symptoms (3.5 on a scale of 0 to 5) and were ELISA and RT-PCR positive, whereas ‘Rojo Pasión’ replicates did not show sharka symptoms and were neither ELISA nor RT-PCR positive. Two replications were assayed for each treatment. Leave samples from each replication were frozen in liquid nitrogen and stored at -80°C, and total RNA was extracted using the Rneasy Plant Mini Kit^®^ (Qiagen, Hilden, Germany). The quality and quantity of total RNA samples were assessed using a NanoDrop^®^ 2000 spectrophotometer (Thermo Scientific, Wilmington, USA) and normalised at the same concentration (5μg, 200 ng/μl). RNA samples were sent to the Scientific Park of Madrid (Spain) (http://www.fpcm.es/es/servicios-a-la-id/servicios/genomica) for library preparation and RNA sequencing. The cDNA libraries were sequenced using an Illumina HiSeq2000 machine to perform 100 paired-end sequencing.

### Bioinformatic analysis

A quality control was performed for the RNA-Seq reads using FastQC software. Pre-processing of the reads was performed with the fastx-toolkit (http://hannonlab.cshl.edu/fastx_toolkit/) in order to filter low quality regions. High quality reads were mapped to the *P*. *persica* genome v1.0 obtained from the Genome database for Rosaceae (GDR, http://www.rosaceae.org/peach/genome) [33] using Tophat 1.4.0 [34] and Bowtie 0.12.7 [35]. Variant calling analysis from read mapping files was performed using Samtools 0.1.18 [36]. High quality variants were obtained after filtering SNPs with quality scores > 20 and INDELs with scores > 50. Differential gene expression (DE) was calculated with the program Cufflinks 1.3.0 [37]. The resulting lists of differentially expressed isoforms were filtered by log2 (log fold change) >2 and < -2 and a q-value <0.05. FPKM values were used to normalise and quantify the gene expression level. Finally, the analysis of Biological Significance was based on Gene Ontology [38] using AgriGO [39] and GeneCodis [40].

### qPCR analysis

To validate the RNA-Seq analysis, qPCR was performed in a new experiment with new plant materials. Total RNA was extracted from leaves using the RNeasy Plant Mini Kit^®^ on three control (Rc and Zc) and inoculated plants (Ri and Rc). Reverse transcription was conducted using the PrimeScript^®^ Reverse Transcriptase Kit (Invitrogen, Applied Biosystems, Madrid, Spain). Eight genes specifically expressed in the different treatments with q-values < 0.05 and high FPKM values were selected to validate RNA-Seq data (S2 Table). Gene-specific primers were designed using Primer3Plus [41]. qPCR reactions were performed in a LightCycler 480 System (Roche Diagnostics, Basel. Switzerland) using SYBR Green to monitor cDNA amplifications. For each primer pair and each sample, the PCR efficiencies (E) and the quantification cycle (Cq) were assessed using LinRegPCR software version 2014.2 [42]. Amplicon specificity was examined by analysis of the melting curve. Relative gene expression (RGE) for a gene of interest (GOI) was calculated using the modified equation E_GOI_^^(CqGOI)^ / E_REF_ ^^(-CqREF)^ from Pfaffl [43]. Three independent biological replicates with at least two technical replicates were performed for each sample. The Cq for the reference normalisation factor (denoted as REF) was calculated by taking three reference genes: peach 18S rRNA [44]; *actin*; and *expansin* [45]. To test the effect of the cultivar and the infection, a one-way analysis of variance (ANOVA) was performed. Means were separated by Fisher's protected LSD test at *p* < 0.05. The analysis was performed with Statgraphics Plus 5.1 Software (Manugistics Inc. Rockville, MD, USA). Correlations between different FPKM values from RNA-Seq and RGE values from qPCR were calculated using the Pearson correlation coefficient.

## Results and Discussion

### RNA-Seq transcriptome profiles

A total of 1,209 million raw pair-end reads of 100 bp in length were generated from the seven samples analysed (Table 1). These raw reads were trimmed by removing adaptor sequences, empty reads, and low quality sequences. As a result, a total of 1,163 million high quality reads (96%), designated as clean reads, were generated in the experiment.

**Table 1:** Mapping characteristics of ‘Rojo Pasión’ (resistant to PPV) and ‘Z506-7’ (susceptible to PPV) apricot genotypes and PPV reads to the reference peach genome (*P*. *persica* v 1.0) and the assayed PPV genome (GenBank: KJ849228.1), in the biological replications of the four samples assayed using RNA-Seq.

Samples	Raw reads	Clean reads	Reads mapped onto the *P*. *persica* v 1.0	Reads mapped onto the PPV genome
**‘Z506-7’ control-1 (Zc-1)**	178,863,722	172,167,692 (96%)	121,897,300 (70.3%)	0 (0.0%)
**‘Z506-7’ control-2 (Zc-2)**	184,354,652	177,433,648 (96%)	127,044,113 (71.7%)	0 (0.0%)
**‘Z506-7’ inoculated-1 (Zi-1)**	176,063,990	169,306,217 (96%)	114,750,138 (67.4%)	8,416,611 (4.9%)
**‘Z506-7’ inoculated-2 (Zi-2)**	175,018,826	168,574,941 (96%)	108,567,566 (64.3%)	10,453,472 (6.2%)
**‘Rojo Pasión’ control-1 (Rc-1)**	146,915,604	141,354,468 (96%)	92,423,368 (65.2%)	0 (0.0%)
**‘Rojo Pasión’ inoculated-1 (Ri-1)**	183,145,536	175,799,070 (95%)	124,637,608 (70.8%)	0 (0.0%)
**‘Rojo Pasión’ inoculated-2 (Ri-2)**	165,533,058	158,922,500 (95%)	112,178,376 (70.8%)	0 (0.0%)
**Total**	1,209,895,388	1,163,558,536 (96%)	801,498,469 (68.8%)	18,870,083 (1.6%)

By iterative alignment, an average of 68.8% of the clean reads were successfully mapped to the v1.0 peach reference genome (http://www.rosaceae.org/), confirming the important level of synteny between *Prunus* genomes [46] and the utility of the peach genome as reference in RNA-Seq studies in different *Prunus* species. Of the unmapped reads, a total of 18.87 million (5.6%) reads derived from the two ‘Z506-7’ apricot samples inoculated with PPV and showing sharka symptoms mapped to the PPV genome (GenBank: KJ849228).

Percentages of mapped RNA-Seq reads in apricot obtained in this work were similar to the 70% mapped reads reported after the apricot transcriptome was explored in similar genotypes using a single 35 nt sequencing protocol [29] but lower than the 89% and 85% reported in peach by Wang et al. [47] and Rubio et al. [31], respectively. However, the mapping percentages in this study were higher than the 50% mapped reads previously reported in Japanese apricot (*P*. *mume* Sieb. et Zucc.) [48].

### SNPs identification

RNA-Seq clean reads from the four treatments in this study were used for Single Nucleotide polymorphism (SNP) and insertion/deletion (INDEL) identification in transcribed regions using the reported peach genome as a reference. A total of 283,057 and 293,565 variations were identified in the transcribed regions (exons) of ‘Rojo Pasion’ and ‘Z506-7’, respectively. Out of these variations, 277,792 were SNPs (98%) and 5,266 were INDELs (2%) in ‘Rojo Pasión’ and 287,626 were SNPs (98%) and 5,939 were INDELs (2%) in ‘Z506-7’. The SNP density was one SNP per 1.0 kb. The highest SNP density was detected in scaffold 1, with 63,140 SNPs identified in the resistant genotypes and 65,402 SNPs in the susceptible genotypes. Scaffold 1 was followed by scaffolds 6 and 4, with more than 30,000 SNPs identified in each region (Table 2). We also identified 124 SNPs specific to the resistant genotype ‘Rojo Pasión’ in the *PPVres* region in scaffold 1 (region of 196 kb) (S3 Table). These SNPs can be used to identify candidate genes that may be responsible for PPV resistance.

**Table 2:** Total and INDEL SNPs identified using RNA-Seq in the two apricot genotypes analyzed ‘Rojo Pasión’ (resistant to PPV) and ‘Z506-7’ (susceptible to PPV) and number of SNPs identified in the main genome regions described as responsible for PPV resistance in apricot by different authors.

	‘Rojo Pasión’	‘Z506-7’	‘Rojo Pasión’ *vs*. ‘Z506-7’
**Total SNPs**	283,057	293,565	—
**Region I (*PPVres*) (194,804 bp) (scaffolld 1: 8,050,804 to 8,244,925)**	325	289	90
**Region II (4,164,415 bp) (scaffolld 1: 8,244,926 to 12,409,341)**	5,740	6,278	—
**Region IV (1,823,665 bp) (scaffolld 5: 12,665,818 to 14,489,483**	4,142	5,561	—
**INDELs**	5,266	5,939	—

One of the 124 SNPs identified in the resistant genotype ‘Rojo Pasión’ in position 8,232,989 of the peach reference genome agrees with the findings of Zuriaga et al. [22], who also identified SNPs in this region after the whole genome sequencing (WGS) of nine different resistant and susceptible apricot genotypes. In addition, another seven SNPs were identified in ‘Rojo Pasión’ with a difference of just one bp with respect to the data of Zuriaga et al. [22] in positions 8,080,435; 8,081,455; 8,097,511; 8,099,699; 8,106,977; 8,115,154; and 8,133,375 respectively. However, none of the 124 specific SNPs matched with the two SNPs identified in the positions 8,156,254 and 8,157,485 of the resistant genotype ‘Lito’ used in MAS for PPV resistance by Decroocq et al. [4]. In addition, only one specific SNP identified in position 11,597,304 (specific of the resistant cultivar ‘Rojo Pasión’) coincided with the candidate SNP proposed by Mariette et al. [49] after genome-wide association analysis of 72 apricot accessions resistant and susceptible to PPV.

Recently a powerful and flexible tool for SNP analysis using SNPlex^™^ high-throughput genotyping technology has been tested in apricot [50]. This technology makes it possible to quickly and easily analyse individual SNPs in apricot genotypes, and it could be used for the genotype and progeny screening of the 124 potential SNPs described in the present work.

### Host transcriptional changes in resistant and susceptible apricot genotypes PPV infected

**Fig 1:**
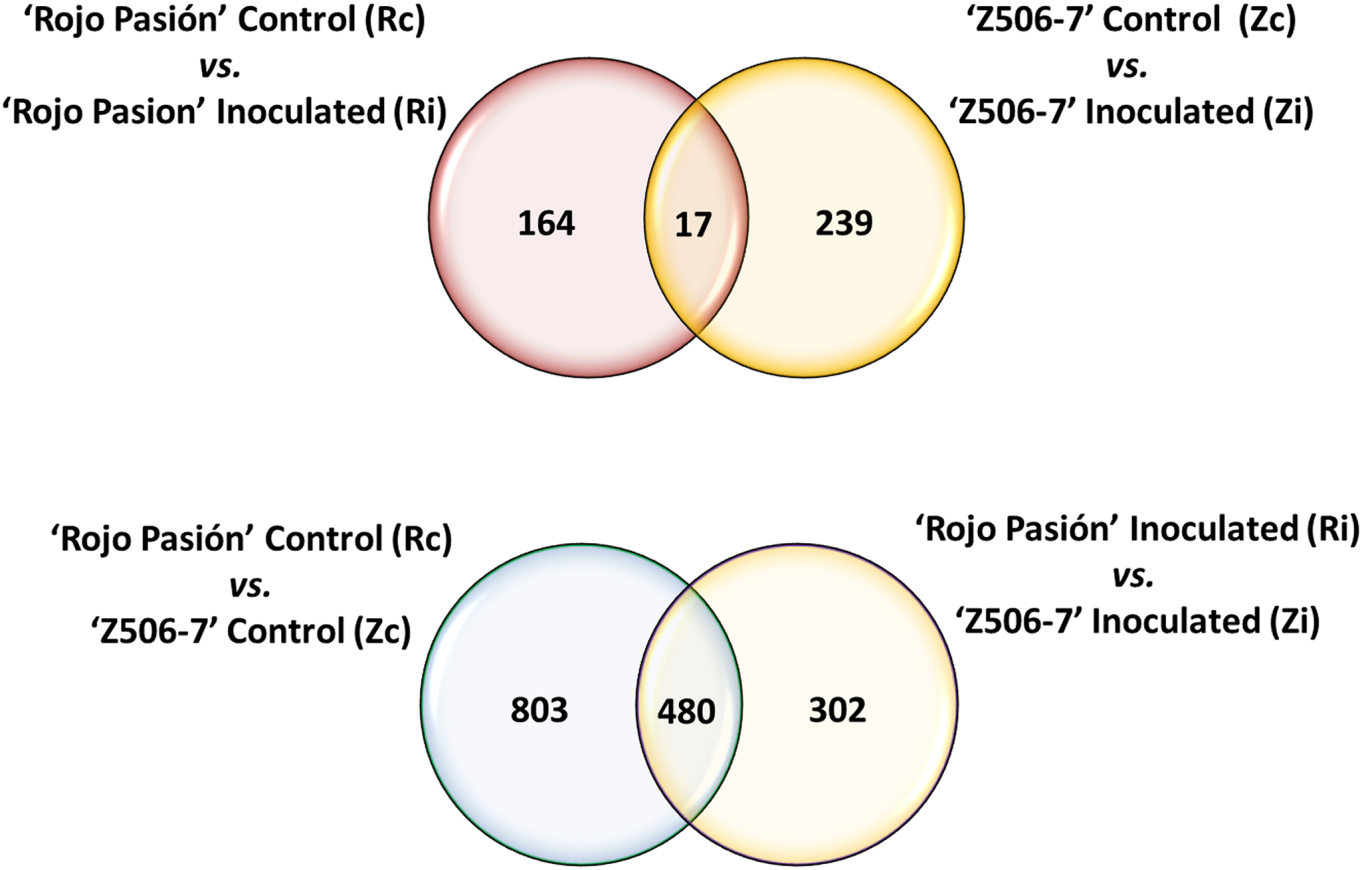
Venn diagram of filtered differentially expressed genes among the four comparisons performed between the PPV resistant apricot genotype ‘Rojo Pasión’ and the PPV susceptible genotype ‘Z506-7’, control and inoculated.

A total of 13,153 differentially expressed genes (DEGs) were identified in the following comparisons: i) ‘Rojo Pasión’ control *vs*. ‘Rojo Pasión’ inoculated (Rc *vs*. Ri); ii) ‘Z506-7’ control *vs*. ‘Z506-7’ inoculated (Zc *vs*. Zi); iii) ‘Rojo Pasión’ control *vs*. ‘Z506-7’ control (Rc *vs*. Zc); and iv) ‘Rojo Pasión’ inoculated *vs*. ‘Z506-7’ inoculated (Ri *vs*. Zi). After filtering the data (fold change ≥ 2 or ≤ -2 and *q*-val < 0.05) a total of 2,005 DEGs were observed in the four comparisons performed. In addition, a total of 164, 239, 803 and 302 filtered DEGs were specific for Rc *vs*. Ri, Zc *vs*. Zi, Rc *vs*. Zc and Ri *vs*. Zi, respectively (Table 3; Fig 1; S4 Table).

**Table 3:** Total and filtered differentially expressed genes (DEGs) from the four apricot samples assayed [‘Rojo Pasión’ (resistant to PPV) control and inoculated and ‘Z506-7’ (susceptible to PPV) control and inoculated] in the four comparisons performed.

	Total DEGs	Filtered DEGs
**‘Z506-7’control *vs*. ‘Z506-7’ inoculated**	1,980	256
**‘Z506-7control’ *vs*. ‘Rojo Pasión’ control**	7,488	1,283
**‘Z506-7’ inoculated *vs*. ‘Rojo Pasión’ inoculated**	2,945	782
**‘Rojo Pasión’ control *vs*. ‘Rojo Pasión’ inoculated**	740	181

Filtering the DEGs increased the detection power for moderately to highly expressed genes, although a loss of power to detect lowly expressed genes has been observed with this method [51]. In contrast, total DEG analysis has the disadvantage that it is not clear whether genes with low read counts are very lowly expressed genes or methodological noise [52]. To confirm the low expression of the genes of interest identified in the total DEG analysis, it is necessary to verify the expression level by qPCR.

Microarray analysis has also been used to study gene expression changes in compatible and incompatible genotypes of melon plants infected with *Watermelon mosaic virus* (WMV, *Potexvirus*). González-Ibeas et al. [53] showed that transcriptomic remodelling due to WMV infection appears to have more profound effects on the resistant genotype than on the susceptible genotype. In a similar vein, regarding the compatible response (susceptibility), Rubio et al. [31] described the differential expression of 1,554 genes in the susceptible peach ‘GF305’ after PPV infection. In model plants, Babu et al. [54] identified more than 2,000 significantly induced genes and 1,457 significantly repressed genes in *Arabidopsis* after PPV infection. Dardick [55] also described 744 differentially expressed genes after PPV infection in *Nicotiana*. In terms of the incompatible response (resistance), Rodamilans et al. [30] described 3,020 differentially expressed genes in the hypersensitive resistant plum genotype ‘Jojo’ after PPV inoculation.

### Functional analysis of differentially expressed genes in resistant and susceptible apricot genotypes

**Fig 2:**
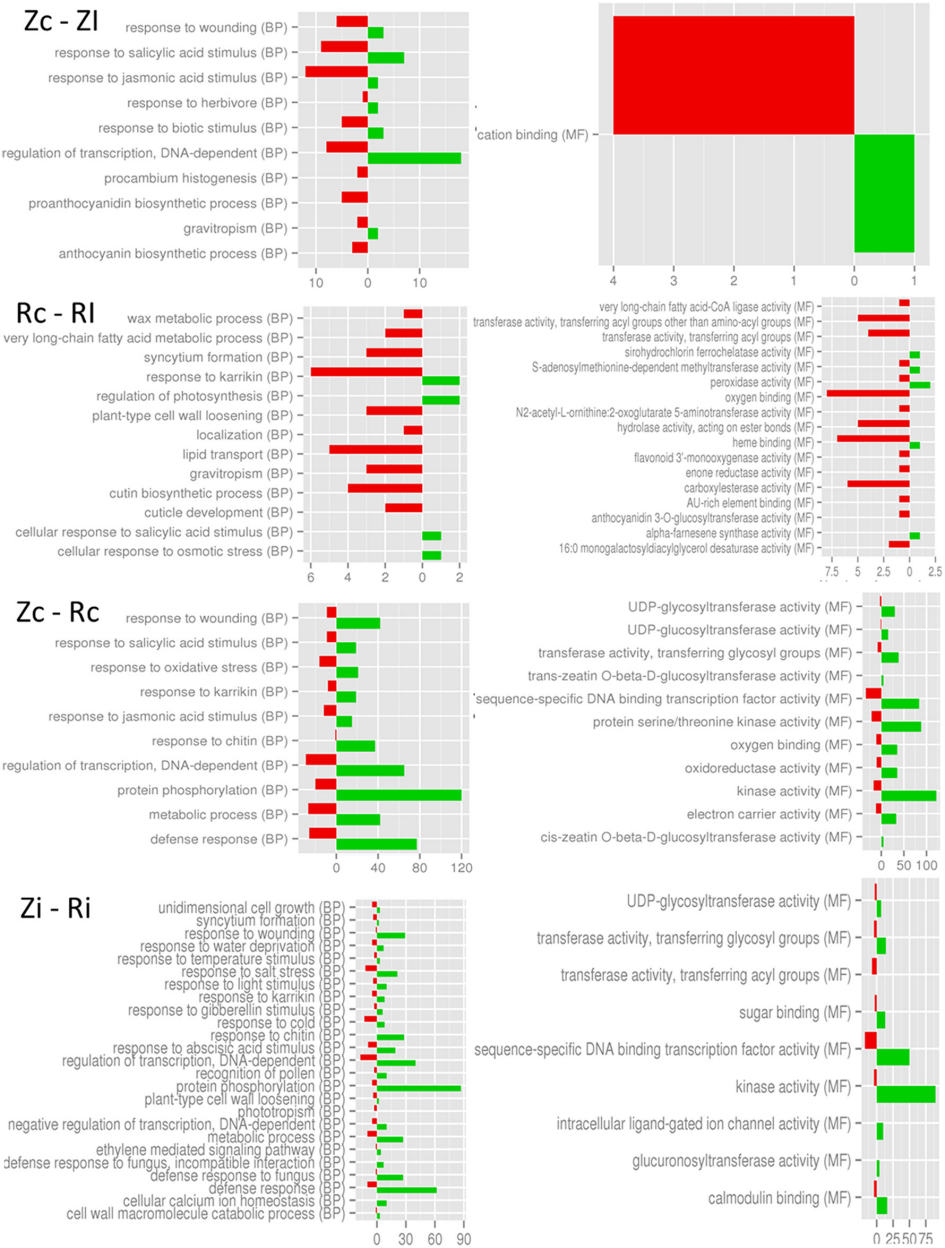
Significant GO annotations of differentially expressed genes involved in biological processes (BP) and molecular functions (MF) in the four comparisons performed between the PPV resistant apricot genotype ‘Rojo Pasión’ and the PPV susceptible genotype ‘Z506-7’, control and inoculated. Number of transcript (abscissa) in the different categories (ordenate) upregulated (green) and upregulated (red).

The 2,502 filtered DEGs identified in the comparisons performed between control and inoculated ‘Rojo Pasión’ and ‘Z506-7’ apricot genotypes were assigned with one or more GO terms. GO assignments fell into broad categories for two of the three major GO functional domains, biological processes (BP) and molecular function (MF) (Fig 2). To elucidate the key processes that were altered in the ‘Z506-7’ genotype during the infection process, we searched for functional enrichment categories in the set of differentially expressed genes.

This analysis showed that DEGs were mainly involved in biological processes associated with the response to different stimuli such as jasmonic acid, salicylic acid, wounding, herbivore attack, biotic stimulus and anthocyanin and proanthocyanin biosynthesis processes (Fig 2).

Only the molecular function ‘cation binding’ was over-represented in the comparison between the control and infected susceptible genotype samples (Zc *vs*. Zi; Fig 2). In the comparison between control and infected resistant genotype samples (Rc *vs*. Ri), processes related to different pathways such as the following were found to be over-represented: the metabolism of terpenoids and polyketides, flavonoid and anthocyanin biosynthesis, porphyrin and chlorophyll metabolism and fatty acid metabolism.

Regarding, biological processes and molecular function analysis, similar results were observed in apricot infected with sharka, where DEGs were related to the response to biotic stimuli, to lipid and carbohydrate metabolism and to the negative regulation of catalytic activity [31]. In addition, Wang et al. [56] found an altered expression of genes in peach leaves involved in defence, cellular transport and protein synthesis and of proteins with a binding function after infection with PPV. DEGs in the infected resistant genotype, compared to the non-infected resistant genotype, were mainly involved the formation of the external cuticule, the cutin biosynthesis process, the wax metabolic process and lipid transport. In the comparison between the susceptible and resistant genotypes, processes related to the defence response and the response to chitin and to wounding were found to be over-represented in the susceptible genotype, in both control and infected samples. It seems that these biological processes are already active in the resistant genotypes.

Furthermore, genes related to secondary metabolism were also found to be over-represented in *Arabidopsis* after PPV infection [54]. These genes were mainly associated with the metabolism of soluble sugar, starch and amino acid; intracellular membrane/membrane-bound organelles; chloroplast; and protein fate.

Functional enrichment analyses were also consistent with the hypothesis that biotic stress marks a transition from growth and reproduction to a physiology and metabolism tailored for defence responses [57]. In general, infected apricot leaves have a low representation of genes involved in the “cellular process” and a high representation of genes implicated in “catalytic activities” and the “regulation of metabolic and biological processes”.

Finally, regarding the behaviour of the resistant genotype ‘Rojo Pasión’, Schurdi-Levraud et al. [27] described a differential expression of genes coding for protein involved in metabolism, signal transduction, defence, stress and intra/intercellular connections after the PPV inoculation of the partially resistant genotype ‘Goldrich’.

### Identification of genes involved in PPV susceptibility and resistance in apricot

RNA-Seq data showed that susceptibility to PPV in apricot is a complex process with hundreds of genes involved in a continuous battle between the virus and the plant (Table 3; Fig 1).

Some of the 256 filtered genes differentially expressed after the inoculation of the susceptible ‘Z506-7’ apricot genotype were similar to those observed during the interaction of PPV with a susceptible peach genotype [31]. These key genes related to susceptibility (Zc *vs*. Zi) included the *endoribonuclease Dicer homolog 2a* (ppa020875m); the *allene oxide synthase* (ppa004133m); the *S-adenosylmethionine synthetase 2* (ppa023986m); and the *major MLP (Muscle LIM* protein*)-like protein 423* (ppa012678m) (S4 Table).

The expression of the *Dicer protein 2a*, the *allene oxide synthase* and the *major MLP-like protein 423* were higher in the control ‘Z506-7’ genotype than in ‘Rojo Pasión’ (Rc *vs*. Zc) before PPV inoculation. However, the expression of the *S-adenosylmethionine synthetase 2* was higher in the resistant genotype (S4 Table). Zuriaga *et al*. (2013) reported finding the *S-adenosylmethionine synthetase* gene in the *PPVres* locus. *S-adenosylmethionine* is a general donor of methyl groups in the transmethylation reactions both in cytosol and in chloroplasts and mitochondria. During PPV infection, the expression of the *Dicer protein 2a* and the *major MLP-like protein 423* was higher in the infected susceptible ‘Z506-7’genotype than in the infected resistant ‘Rojo Pasión’ genotype (Ri *vs*. Zi) (see S4 Table).

The differential expression of the *endoribonuclease Dicer protein 2a*, previously described in peach [31] could suggest the suppression of a gene silencing plant response by the virus HCPro and P1 proteins [58]. For example, at the phenotypic level, Karayiannis et al. [6] observed a reduction in PPV symptoms in susceptible apricot genotypes in the second cycle of study after a first cycle of clear infection and symptom development. These authors attributed this response to a silencing of the virus.

Resistance to PPV in apricot is also a complex mechanism based on an interaction between the host and pathogen in which different genes are involved (Table 3; Fig 1). The *PPVres* locus contains a total of 31 predicted transcripts [22], and some of these key genes were differentially expressed in the resistant ‘Rojo Pasion’ apricot genotype after PPV inoculation (comparison Rc *vs*. Ri). These genes included ppa000229 (*transporter protein*); ppa013116 (*unknown protein*); and ppa022195 (*TRAF-like family protein*) (S4 Table).

Differential expression of the *transporter protein* and the *TRAF-like family protein* was observed both when comparing the genotypes ‘Rojo Pasión’ and ‘Z506-7’ before (Rc *vs*. Zc) and after PPV inoculation (Ri *vs*. Zi). The level of differential expression was higher in the resistant genotype (S4 Table).

**Fig 3:**
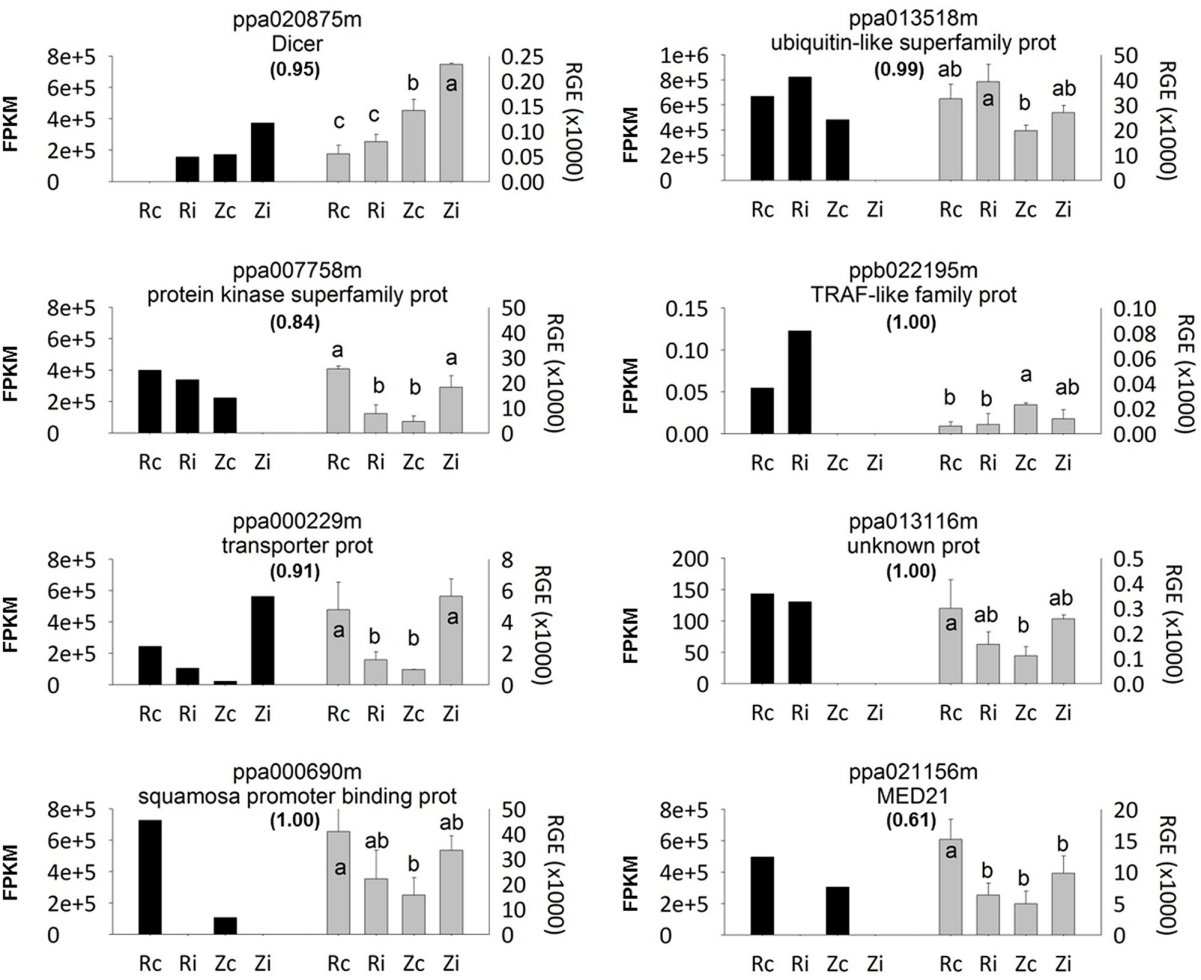
qPCR expression analysis for candidate genes selected from the RNA-Seq analysis, showing FPKM values in black. Relative gene expression (RGE) (in grey) in the four samples assayed: the resistant ‘Rojo Pasión’ genotype, control (Rc) and inoculated (Ri), and the susceptible ‘Z506-7’ genotype, control (Zc) and inoculated (Zi). Error bars represent the standard error for the three independent biological replicates. Different letters indicate significant differences among the samples according to the LSD test with a *p*-value of 0.05. Between brackets Pearson correlation coefficients between FPKM (fragments per kilobase pair of transcript per million mapped reads) values from RNA-Seq and RGE values from qPCR.

The described results confirm that *MATH* genes (which control the long-distance movement of viruses) are involved in resistance, but probably together with other genes (effect of the genetic background). Along these lines, Decroocq et al. [4] developed a single length polymorphism (SSLP) marker called ZP002, which is based on the 5-bp deletion present in the EST ppb0022195, at map position 8,157,652. This EST encodes a *TRAF-like protein* that was proposed as a candidate gene by Zuriaga et al. [22]. Among the four *TRAF-like proteins* (ppa022254, ppb022195, ppb020867 and ppa019595) tested in the RNA-seq analysis, we could only validate ppb022195 by qPCR due to the low expression of the other three proteins (Fig 3).

Despite the fact that the allele with the deletion in resistant genotypes (112 bp) was also detected in ‘Rojo Pasión’ by RNA-seq, the level of expression was really low. This data could indicate that the gene responsible for PPV resistance in apricot presents a low level of expression. These results also reinforce the hypothesis of a second locus involved in the control of this resistance [4,5].

In addition, other genes may participate in the complex process of the incompatible PPV/apricot response (resistance). In this vein, Rubio et al. [59] suggested that PPV resistance is polygenic in nature in peach and related species such *Prunus davidiana* and that the genetic background of the parents plays a key role in this resistance. In this sense we can remark the differential expression (Rc *vs* Ri) of *Pleiotropic drug resistance 9* gene (ppa000229) (scaffolld 1: 8,128,553) inside the *PPVres* region described as an important resistant gene [60] and CAP (*Cysteine-rich secretory proteins*, *Antigen 5*, *and Pathogenesis-related 1 protein*) superfamily protein (scaffolld 1: 6,426,865) (S4 Table) described near the *PPVres* region also described as an important resistant gene [61]. This region of scaffold 1 has been also described as responsible of PPV resistant in different QTL analysis assays [10,11]. In addition, we can remark the differential expression (Rc *vs* Ri) of LEA (*Late embryogenesis abundant protein*, ppa008651m) (scaffolld 1: 7,990,165) proteins located near the *PPVres* region in the mentioned region [10,11] that are more expressed in Ri than in Rc (see S4 Table). These proteins have been described as important resistant proteins [62,63].

The observed resistant response in apricot is different from the response observed in plum resistance through hypersensitivity. Rodamilans et al. [30] identified more than 3,000 differentially expressed unigenes after the PPV inoculation of the resistant European plum genotype ‘Jojo’, which developed a hypersensitive response. Out of these identified unigenes, 154 were characterised as potential resistance genes, and 10 codified NBS-LRR proteins. These authors hypothesised that the nature of PPV resistance in this species is related to the *NBS-LRR* genes. In apricot, however, the candidate gene analysis of these genes did not provide satisfactory results [12,24,25].

### Validation of gene expression profiles using qPCR

The expression trends of eight selected genes were similar to the results obtained with RNA-Seq analysis (Fig 3). Pearson correlation coefficients between fragments per kilobase pair of transcript per million mapped reads (FPKM) values from RNA-Seq and relative gene expression (RGE) values from qPCR of the assayed genes ranged between 0.61 (ppa021156m) and 1.00 (ppb022195m, ppa013116m and ppa000690) with an average value of 0.91.

The expression of the genes coding for the *protein kinase superfamily protein* (ppa007758m), the *transporter protein* (ppa000229m), and the *MED21* (ppa021156m) was lower in PPV-inoculated leaves compared to the non-inoculated leaves of the resistant genotype ‘Rojo Pasión’ (Fig 3).

Regarding the susceptible full-sib genotype, the expression of several genes, including the *Dicer protein 2a* (ppa020875), *protein kinase superfamily* (ppa007758m) and the *transporter protein* (ppa000229m), was higher in leaves from the susceptible genotype ‘Z506-7’after PPV inoculation compared to the non-inoculated leaves. However, similar expression was detected in the control and inoculated ‘Z506-7’ leaves for the genes encoding for the *ubiquitin-like superfamily* (ppa013518); the *TRAF-like family protein* (ppb022195); the unknown protein (ppa013116m); the *squamosa promoter binding protein* (ppa000690); and *MED21* (ppa021156m) (Fig 3).

Gene expression analysis using qPCR highlighted the over-expression of the gene coding the *Dicer protein 2a* in the susceptible genotype, indicating the suppression of a gene silencing mechanism of the plant through PPV HCPro and P1 PPV proteins. In addition, in the case of the resistance genotype, results showed the expression of genes from the homology *domain MATH* (including the *TRAF-like family protein*) inside the *PPVres* region involved in the resistance to PPV in apricot. *TRAF-like family protein* (ppb022195) has also been recently proposed as candidate resistant gene by Mariette et al. [49] after genome-wide association analysis of resistant and susceptible apricot genotypes.

Finally, the different expression levels detected between RNA-Seq and qPCR results could be due to the low expression level of the genes analysed and the bioinformatics biases, which include alignment choices, estimation of transcript expression, etc. [64]. In addition, other factors affecting this discrepancy include the dynamic nature of the transcriptome [65]. This dynamism is greater in the case of susceptible genotypes when the virus is present, which greatly increases the range of the plant response in accordance with the accumulation of the virus and the presence of disease symptoms.

## Conclusions

From the methodological point of view, RNA-Seq has proven to be a very powerful tool in the analysis of the PPV/apricot interaction, although it is not the definitive tool for solving the gene expression base and the genomic determinants of this trait. This technique is nevertheless an important complementary tool to other means of studying genomics that produce lesser amounts of data, static behaviour and clearer polymorphisms between genotypes. An integrated approach should compensate for the main disadvantages of using RNA-Seq as an analysis tool, such as the high amount of data, the dynamism of transcriptome expression, and the complexity of regulation. In this study, transcriptomic differences at the gene expression level confirmed that susceptibility to PPV in apricot is a complex process based on a continuous battle between the virus (PPV) and the plant (*Prunus*) at the pathogen resistance gene level (*allene oxide synthase*, the *S-adenosylmethionine synthetase 2* and the *major MLP-like protein 423*) and gene silencing level. This response is similar to the response observed in peach. On the other hand, resistance to PPV in apricot is also a complex process that could involve *MATH* genes (which control the long-distance movement of viruses). Furthermore, other genes inside (*Pleiotropic drug resistance 9* gene) or outside (CAP, *Cysteine-rich secretory proteins*, *Antigen 5 and Pathogenesis-related 1 protein*; and LEA, *Late embryogenesis abundant protein*) *PPVres* region could also be involved in the resistance.

## Supporting Information

S1 Table SSR genotyping of the apricot genotypes assayed: ‘Rojo Pasión’ (resistant to PPV) and ‘Z506-7’ (susceptible to PPV). (XLSX)

S2 Table Primer sequences representing a set of eight genes differentially expressed in the different treatments and three internal controls genes selected for qPCR. (XLSX)

S3 Table Total SNPs (Single nucleotide polymorphisms) and insertions/deletions (INDELs) identified by RNA-Seq in the apricot genotypes assayed, ‘Rojo Pasión’ (resistant to PPV) and ‘Z506-7’ (susceptible to PPV). Different SNPs identified in the *PPVres* loci of ‘Rojo Pasión’ (green) and ‘Z506-7’ (red) are also indicated.
(XLSX)

S4 Table Total and filtered [ln (fold_change) >2 and < -2 and a q-value <0.05] genes differentially expressed in control and inoculated ‘Rojo Pasión’ (Rc *vs*. Ri); control and inoculated ‘Z506-7’ (Zc *vs*. Zi); control ‘Rojo Pasión’ and ‘Z506-7’ (Rc *vs*. Zc); and inoculated ‘Rojo Pasión’ and ‘Z506-7’ (Ri *vs*. Zi). (XLSX)
